# Costs of oropharyngeal squamous cell cancer treatment in Finland

**DOI:** 10.1007/s00405-023-08124-4

**Published:** 2023-07-24

**Authors:** Juhana Tikkanen, Teija Nieminen, Patrik Lassus, Mikko Tenhunen, Lasse Lehtonen, Antti Mäkitie

**Affiliations:** 1 Department of Otorhinolaryngology–Head and Neck Surgery, University of Helsinki and Helsinki University Hospital, P.O. Box 263, 00029 HUS Helsinki, Finland; 2 Department of Perioperative and Intensive Care Medicine, University of Helsinki and Helsinki University Hospital, Helsinki, Finland; 3 Department of Plastic Surgery, University of Helsinki and Helsinki University Hospital, Helsinki, Finland; 4 Comprehensive Cancer Center, University of Helsinki and Helsinki University Hospital, Helsinki, Finland; 5 HUS Diagnostic Center, University of Helsinki and Helsinki University Hospital, Helsinki, Finland; 6 Research Program in Systems Oncology, Faculty of Medicine, University of Helsinki, Helsinki, Finland; 7 Division of Ear, Nose and Throat Diseases, Department of Clinical Sciences, Intervention and Technology, Karolinska Institute and Karolinska University Hospital, Stockholm, Sweden

**Keywords:** Head and neck cancer, Surgery, Radiotherapy, Chemotherapy, Outcome, Microvascular reconstruction

## Abstract

**Background:**

Oropharyngeal squamous cell carcinoma (OPSCC) can be treated with definitive (chemo)radiotherapy ((C)RT) or primary surgical treatment (PST) with or without postoperative oncologic treatment. The prognosis of OPSCC does not essentially depend on the treatment modality, which allows to consider secondary decision-making aspects such as treatment costs when recommending an individual treatment modality. We attempted to analyze the costs associated with definitive (C)RT and PST in the treatment of OPSCC in Finland.

**Materials and methods:**

We included 73 patients diagnosed with OPSCC at the Helsinki University Hospital (HUS) (Helsinki, Finland) in 2019 and 2020. Treatment costs were defined as the costs incurred in the specialized medical care during the first 12 months after the diagnosis was established.

**Results:**

Definitive RT and definitive CRT were on a 1-year horizon associated with median costs of approximately 10 700€ and 13 300€, respectively; while, the median costs of PST equaled about 40 600€. The costs of definitive (C)RT mostly consisted of the costs of (chemo)radiotherapy sessions; while, the operating room costs and the costs of intensive care and stay on a ward drove the costs of PST.

**Conclusions:**

PST is associated with 2–3 times higher median costs than definitive (C)RT in Finland. The finding differs from the results previously reported in North America, which is related, e.g., to differences in the treatment practices as well as in the regulation of the health care systems.

## Introduction

Head and neck cancers (HNCs) form the seventh most common cancer type globally [5]. The incidence of many subtypes of HNCs has decreased during the past few decades, which is most likely caused by the decrease in the tobacco and alcohol consumption [13]. However, oropharyngeal squamous cell carcinoma (OPSCC) provides an important exception from the positive overall development: its incidence has remarkably increased in the twenty-first century, following a rise in the cancers related to the human papillomavirus (HPV) [3]. Internationally, the share of HPV positive OPSCCs rose from 32 to 53% between 1995 and 2015 [13]. In the United States, OPSCC has overtaken cervical cancer as the HPV-related cancer type with the highest number of new annual cases [2]. Despite the emergence of HPV vaccines, the incidence of OPSCC has been estimated to start to decrease only in the early 2060s as the latency period between exposure to HPV and the development of OPSCC can extend up to 30 years [1]. Approximately, 200 new annual cases of OPSCC are nowadays diagnosed in Finland [11].

The treatment of OPSCC can be based on either definitive (chemo)radiotherapy ((C)RT) or primary surgical treatment (PST) with or without postoperative (C)RT. The choice of treatment modality depends, e.g., on the T and N classes of the disease. A small and local carcinoma can be treated with a single modality; whereas, the use of a combined approach is applied along with higher T and N classes [4]. The prognosis of OPSCC has been shown not to essentially depend on the chosen treatment modality. For example, studying a sample of 22,000 patients. Chen et al. [4] showed that, after controlling for other factors, the treatment modalities were associated with comparable 5-year overall survival rates. This allows to consider secondary factors, such as treatment costs, when recommending an individual treatment modality, which is an additional decision-making aspect.

Previously, the treatment costs of OPSCC have been analyzed in some North American studies. Tam et al. [14] analyzed data of 15 patients who underwent transoral robotic surgery (TORS) and 15 stage-matched patients treated with CRT. The costs related to TORS were on a 12-month time span approximately 14% lower than the costs of CRT. Building on a simulation model, de Almeida et al. [6] estimated that on a ten-year horizon TORS leads to approximately 3% lower costs than CRT when examining OPSCC patients with a low T stage. Moore et al. [9] found that the costs of CRT were on average higher than the costs of surgery during the first three months of treatment even in cases where postoperative (C)RT was employed.

The afore-mentioned results cannot be directly applied to other countries because of differences in healthcare systems and treatment practices. For instance, the use of TORS in the treatment of OPSCC is still unestablished in Finland. We, therefore, compared the costs of surgery and (C)RT in the management of OPSCC in Finland.

## Materials and methods

In total, 121 patients were diagnosed with OPSCC at the Helsinki University Hospital (HUS) (Helsinki, Finland) in 2019 and 2020. After excluding the patients treated with a non-curative intent (*n = *11) and the patients for whom data on the treatment costs were not available for a sufficiently long time period (*n = *37), the final series consisted of 73 patients (53 males, 20 females, Table 1). Twenty-six patients received definitive RT, 37 definitive CRT, and 10 primary surgical treatment with or without postoperative (C)RT. Tonsils and tongue base were the most common tumor sites forming 92% of all cases. p16 status of the primary tumor was positive for 90% of the 42 patients for whom this information was available.

**Table 1:** Information on the patient sample

	All	Definitive RT	Definitive CRT	PST
Primary treatment modality	73 (100.0%)	26 (35.6%)	37 (50.7%)	10 (13.7%)
Average age, years	63.4	69.6	58.2	66.7
Sex				
Men	53 (72.6%)	21 (80.8%)	24 (64.9%)	8 (80.0%)
Women	20 (27.4%)	5 (19.2%)	13 (35.1%)	2 (20.0%)
Tumor site				
Base of tongue	26 (35.6%)	8 (30.8%)	15 (40.5%)	3 (30.0%)
Tonsil	41 (56.2%)	16 (61.5%)	20 (54.1%)	5 (50.0%)
Other	6 (8.2%)	2 (7.7%)	2 (5.4%)	2 (20.0%)
p16 classification of the primary tumor				
Positive	38 (52.1%)	15 (57.7%)	22 (59.5%)	1 (10.0%)
Negative	4 (5.5%)	2 (7.7%)	0 (0.0%)	2 (20.0%)
Unknown	31 (42.5%)	9 (34.6%)	15 (40.5%)	7 (70.0%)

The financial data for the study were obtained from the financial services of the HUS. In this study, treatment costs are defined as the costs accrued during the first 12 months following the diagnosis, reflecting the fact that a vast majority of the total treatment costs are accumulated during this time period. Although this definition excludes the costs of diagnostic tests performed before the diagnosis was confirmed, this is not problematic as such costs are irrelevant when the purpose is to analyze treatment costs. The treatment costs of a recurrent disease are considered to the extent that these costs were observed within one year of the initial diagnosis.

The financial data provided by the financial services of the HUS divide the total costs into eight subcategories: outpatient costs, inpatient costs, operating room costs, outpatient procedure costs, medical imaging costs, laboratory costs, pathology-related costs, clinical physiology-related costs, as well as other unspecified costs. Table 2 discusses in detail the nature of the costs belonging to each of the nine cost subcategories.

**Table 2:** Information on the cost categories

Cost category	Description
Outpatient costs	Outpatient costs consist, e.g., of the costs of pre-treatment planning visits and post-treatment follow-up visits at the outpatient clinic for patients with HNC; in 2020, for example, the cost of a single visit was 133€. In addition, the costs of RT and CRT sessions are in the financial data partly classified as outpatient costs. For instance, about 20% (40€) of the total cost of a RT session (179€ in 2020) is categorized as outpatient costs; the respective share for the cost of a single CRT session (230€ in 2020) is approximately 50% (114€)
Inpatient costs	Inpatient costs include the bed ward costs but not the costs of intensive care periods. In 2019 and 2020, the cost of one day in the bed ward was about 730€
Operating room costs	The category consists of the costs of the procedures performed in the operating room. The surgical resection of the primary tumor, the reconstruction of the surgical defect with a microvascular flap as well as the dissection of the lymph nodes of the neck serve as examples of operating room procedures
Outpatient procedure costs	RT and CRT sessions, various endoscopic examinations as well as nutritional therapy, speech therapy and physiotherapy are examples of outpatient procedures. In the financial data, the costs of individual RT and CRT sessions are partly divided into outpatient costs and partly into outpatient procedure costs. In 2020, for example, about 80% (139€) of the cost of a radiotherapy session was categorized as outpatient procedure cost; the corresponding share for a chemoradiotherapy session was about 20% (45€)
Medical imaging costs	The CT scan used in radiotherapy treatment planning as well as the ultrasound of the neck are the most common radiological examinations for OPSCC patients, which together make up the medical imaging costs
Laboratory costs	The category is composed of the costs of laboratory tests
Pathology-related costs	Pathology-related costs include the costs arising from the analysis of the samples obtained, e.g., in surgical operations. Since this study examines the costs accrued after the diagnosis has been established, the pathology-related costs accumulated during the pre-diagnosis phase are not considered
Clinical physiology-related costs	The costs in this category mostly consist of the costs of a PET CT scan that is made to some of the patients approximately 3 months after the oncologic treatments have ended
Other costs	First, the category “other costs” includes quantitatively insignificant costs such as the costs of blood products and supplies used in operations. Second, the category contains the costs not separated into their own cost category in the financial data, most importantly the costs of intensive care. Third, approximately 30% of the total cost of a single chemotherapy session (about 230€ in 2020) is considered in this category. To summarize, in the context of PST this category can be interpreted as the costs of treatment in the intensive care unit; in the cost analysis of definitive CRT, this category mainly reflects the costs of chemoradiotherapy sessions not considered elsewhere

## Results

### The costs of definitive radiotherapy

**Fig. 1:**
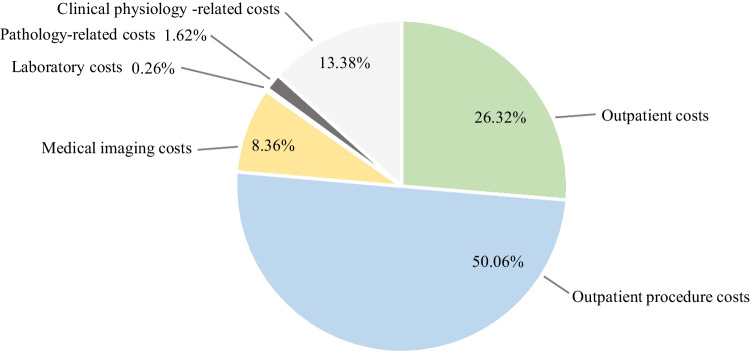
The distribution of the median total costs of definitive RT

Table 3 shows the median and average total costs related to the treatment of the group of patients who received definitive RT as well as the distribution of the total costs into cost categories, which is also visualized in Fig. 1. The median costs presented in Tables 3, 4, 5 represent the median costs of each cost category, and their sum is not equal to the median total costs. To improve comparability, the relative shares of the median costs of the cost categories have been scaled in Tables 3, 4, 5 to sum up to one hundred percent.

**Table 3:** The median and average costs of definitive RT by cost category

	Median costs		Average costs	
Total costs	10 716.79 €	100.0%	13 450.60 €	100.0%
Outpatient costs	2 723.00 €	26.3%	2 799.43 €	20.8%
Inpatient costs	- €	0.0%	1 755.00 €	13.0%
Operating room costs	- €	0.0%	1 077.83 €	8.0%
Outpatient procedure costs	5 177.85 €	50.1%	5 285.42 €	39.3%
Medical imaging costs	865.00 €	8.4%	754.67 €	5.6%
Laboratory costs	27.00 €	0.3%	93.70 €	0.7%
Pathology-related costs	167.40 €	1.6%	234.38 €	1.7%
Clinical physiology-related costs	1 383.85 €	13.4%	964.25 €	7.2%
Other costs	- €	0.0%	485.94 €	3.6%

**Table 4:** The median and average costs of definitive CRT by cost category

	Median costs		Average costs	
Total costs	13 349.22 €	100.0%	14 670.95 €	100.0%
Outpatient costs	3 715.00 €	31.0%	3 954.38 €	27.0%
Inpatient costs	- €	0.0%	636.82 €	4.3%
Operating room costs	- €	0.0%	1 319.68 €	9.0%
Outpatient procedure costs	5 175.00 €	43.1%	5 235.62 €	35.7%
Medical imaging costs	981.00 €	8.2%	998.01 €	6.8%
Laboratory costs	207.67 €	1.7%	239.03 €	1.6%
Pathology-related costs	112.40 €	0.9%	187.14 €	1.3%
Clinical physiology-related costs	1 384.00 €	11.5%	1 180.79 €	8.0%
Other costs	421.48 €	3.5%	919.48 €	6.3%

**Table 5:** The median and average costs of PST by cost category

	Median costs		Average costs	
Total costs	40 574.24 €	100.0%	39 553.95 €	100.0%
Outpatient costs	1 293.70 €	4.0%	1 702.10 €	4.3%
Inpatient costs	6 567.30 €	20.4%	9 764.00 €	24.7%
Operating room costs	10 861.50 €	33.7%	11 231.53 €	28.4%
Outpatient procedure costs	911.50 €	2.8%	2 168.58 €	5.5%
Medical imaging costs	645.19 €	2.0%	590.84 €	1.5%
Laboratory costs	484.04 €	1.5%	447.41 €	1.1%
Pathology-related costs	702.80 €	2.2%	710.84 €	1.8%
Clinical physiology-related costs	17.68 €	0.1%	184.04 €	0.5%
Other costs	10 770.65 €	33.4%	12 754.62 €	32.2%

The median total costs of definitive RT were approximately 10 700€, of which 50% consisted of outpatient procedure costs, mainly radiotherapy. Radiotherapy also explained a part of the outpatient costs as the total cost of one radiotherapy session (e.g., 179€ in 2020) was in the financial data divided between outpatient costs (40€) and outpatient procedure costs (139€). The treatment of OPSCC typically consisted of 30–35 radiotherapy sessions, indicating that the total costs of a radiotherapy period were approximately 5 400–6 300€.

The operating room costs were generally not essential for patients receiving definitive RT and equaled zero for a median patient. For some patients, however, swallowing difficulties caused by radiotherapy established the need for percutaneous endoscopic gastrostomy. In addition, one patient in the sample underwent surgical resection of the primary tumor due to insufficient response to radiotherapy. Since operations and related inpatient periods were expensive compared to radiotherapy sessions, such operations performed on individual patients led the average total costs of definitive RT to be considerably higher than the median total costs of definitive RT.

The costs of clinical physiology formed the third-largest cost category when examining median costs. The costs of PET CT scan, typically performed a few months after the last radiotherapy session, accounted for most of these costs. Imaging costs, e.g., ultrasound and MRI of the neck, formed less than 10% of the total costs of definitive RT.

### The costs of definitive chemoradiotherapy

**Fig. 2:**
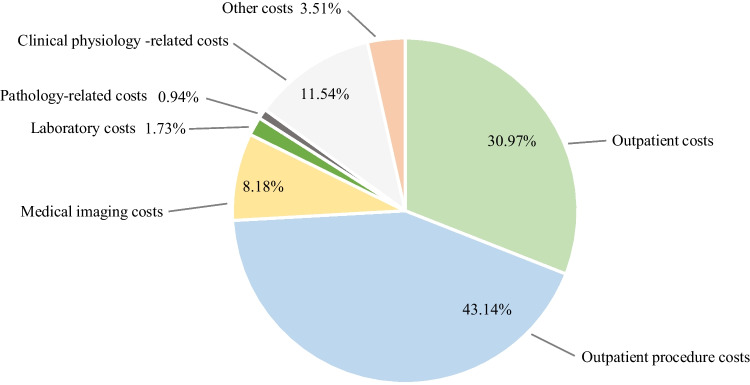
The distribution of the median total costs of definitive CRT

The median and average costs of definitive CRT are shown in Table 4 and Fig. 2. The median total costs of about 13 300€ exceeded the median total costs of definitive RT by approximately 2 600€. The costs of chemotherapy explained most of the difference: in 2020, for example, the cost of one chemotherapy session was approximately 230–240€ and the total cost of the treatment of seven chemotherapy sessions about 1 600–1 700€. Of the median total costs of both definitive RT and definitive CRT, an approximately equal proportion consisted of the costs of the (chemo)radiotherapy sessions, although the share of outpatient costs was higher in the context of definitive CRT due to how the costs of (chemo)radiotherapy sessions were classified in the financial data.

The operating room and inpatient costs were mostly low for patients treated with definitive CRT. Most of the patients receiving definitive CRT did not undergo any operations as indicated by the median operating room costs of zero, although procedures such as dissection of the lymph nodes of the neck and percutaneous endoscopic gastrostomy were performed to some of the patients. The relatively long periods in the bed ward of some individual patients treated with definitive RT led the average inpatient costs to be lower for definitive CRT patients than for definitive RT patients.

The costs of clinical physiology—essentially, the cost of the PET CT scan performed a few months after the treatment—was an important cost category in the group that received definitive CRT as well. The laboratory costs were higher for patients treated with definitive CRT than for those treated with definitive RT, reflecting the fact that use of cisplatin, the most commonly used chemotherapy drug for OPSCC patients in Finland [8], required regular laboratory tests as it can cause bone marrow failure. The median other costs of 420€ were mainly attributable to the share of the chemotherapy costs classified as other costs in the financial data.

### The costs of primary surgical treatment

**Fig. 3:**
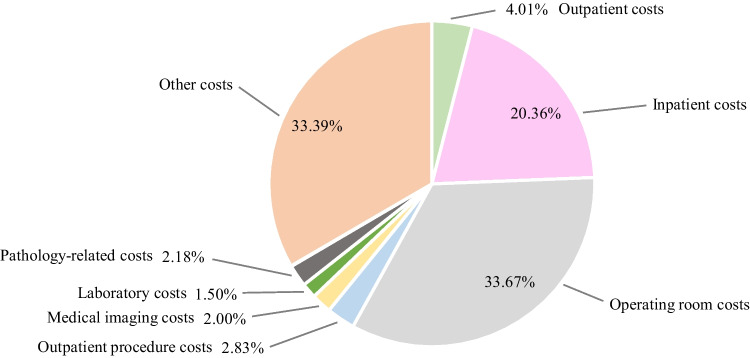
The distribution of the median total costs of PST

Table 5 and Fig. 3 show the median and average costs of PST. The median total costs of PST, approximately 40 600€, were 2–3 times higher compared to the median total costs of definitive RT and definitive CRT. More than 85% of the median total costs were composed of operating room costs, inpatient costs and other costs, which in the context of PST predominantly refers to the costs of intensive care. Given the cost of one day in the bed ward of about 730€ in 2019 and 2020, the median inpatients costs of about 6 600€ implied that a typical patient spent nine days in the bed ward during the first year after the diagnosis, although there was remarkable dispersion between patients in the data. Of the ten patients in the data who received PST, none received postoperative CRT and four received postoperative RT, the costs of which were divided into outpatient procedure costs and to a lesser extent outpatient costs as previously.

## Discussion

Definitive oncologic treatment has during the past decades become more common than primary surgical treatment (PST) both in the U.S. [4] and in Finland [8]. While approximately 85% of Finnish OPSCC patients treated with a curative intent received PST in the late 1990s [10], the corresponding figure was 48% in the early 2000s [8]. We investigated the costs of OPSCC treatment at a large tertiary care academic hospital with a referral area of 2.2 M inhabitants. This population-based series comprises more than one third of these patients in Finland (5.5 M), where the management of HNCs is centralized to the five university hospitals. In Finland, the treatment of HNCs is based on the guidelines given by the Finnish Head and Neck Oncology Working Group, and the treatment of each patient is planned in a multidisciplinary team consisting of otorhinolaryngologists—head and neck surgeons, maxillofacial surgeons, plastic surgeons, and oncologists (www.fshno.fi 2020). We compared the costs of surgical and oncological treatment and found the PST approach being 2–3 times more expensive than definitive oncologic treatment. Since PST and definitive RT are considered alternative treatment options especially for OPSCC patients with a small and local disease [7], it is noteworthy that in our analysis the median operating room costs of PST alone corresponded to the median total costs of definitive RT.

The present findings are inconsistent with previously published North American studies [6, 9, 14], all of which concluded that PST of OPSCC is associated with lower costs. There are several reasons that are likely to contribute to these contradictory findings. First, aspects related to the regulation of the health care systems are likely to play a significant role. In Europe, drug prices are more strictly regulated by authorities than in the U.S., where pharmaceutical companies can more independently decide the prices of their drugs. As a result, the U.S. spent 1 310$ per capita on prescription and over-the-counter medicines in 2020, whereas the corresponding figure, for example, in Finland was 562$ [12]. While Tam et al. [14] did not report the absolute costs of the treatment of OPSCC, the costs of both radiotherapy and chemotherapy sessions, for example, at the Helsinki University Hospital—about 180€ and 230€ in 2020—can be regarded as relatively low. Indeed, the fact that definitive RT and definitive CRT are cheaper treatment modalities at the HUS seems to primarily result from the affordability of oncologic treatment rather than from the expensiveness of surgical treatment in Finland. While there is remarkable variation in the costs of (chemo)radiotherapy between hospitals in Finland, definitive oncologic treatment would remain the cheaper treatment option even assuming significantly higher costs of (chemo)radiotherapy.

Compared to previous studies, the partly different treatment practices of OPSCC also contribute to the opposite results. In the studies by Tam et al. [14], de Almeida et al. [6] and Moore et al. [9], surgical treatment was based on either TORS or TOS. These approaches are less invasive than open surgery that is the predominant operative approach in Finland. Compared to endoscopic methods, open surgery is associated with longer hospitalization periods. In the study by Tam et al. [14], the patients spent on average 5.1 postoperative days on a hospital ward, and inpatient costs formed less than 10% of the total costs of PST. Moore et al. [9] reported an average postoperative stay of 2.4 days. In our analysis, the costs of intensive care and stay on a ward accounted for approximately 50% of the median total costs of PST, which is a consequence of significantly longer postoperative hospitalization periods. In the study by Tam et al. [14], chemotherapy was mainly carried out with cetuximab, that is more expensive than cisplatin that is commonly used in the treatment of OPSCC in Finland [8]. In the study by Moore et al. [9], the costs of definitive CRT were increased by all patients routinely having a percutaneous endoscopic gastrostomy,while in Finland, this is preserved only for a selective group of patients.

In this study, the patient groups treated with alternative methods are not stage matched, meaning that this study rather attempts to describe the costs related to the treatment modalities, given the fact that different patients are treated differently, than to estimate how the costs related to modalities would compare to each other if the patient groups were comparable as in the study by Tam et al. [14]. The fact that the patient groups are not comparable also raises the question of whether the characteristics of the patient groups could explain the higher costs of PST. The median T classification for both the patients treated with definitive RT, definitive CRT and PST is 2,while, the median N classification is 1 for the groups treated with definitive RT and definitive CRT and 0 for the group treated surgically. Therefore, the suggestion that the higher costs of PST could result from a patient population requiring more invasive or extensive treatment is not supported by the data.

The relative affordability of definitive RT and definitive CRT found in this study forms only one aspect in the decision-making in OPSCC treatment planning. While the choice of treatment modality must be primarily based on expectations on its effectiveness as well as on the ability of the patient to tolerate the treatment and potential adverse effects, the impacts on later quality of life must be taken into consideration. In England, for example, the National Institute for Health and Care Excellence has defined society’s willingness to pay per one quality-adjusted life year to be about £20 000–£30 000. Therefore, it could make economic sense to prefer more expensive treatment modalities if they were associated with significantly higher future quality of life. Unfortunately, the data applied in this study do not allow analysis of the impacts of the treatments on the prognosis and future quality of life of the patients.

There are many potential ways to further expand the analysis on the treatment costs of OPSCC. This study follows the approach adopted by Tam et al. [14] by considering the costs accrued during the first 12 months after the diagnosis. Although most of the treatment costs are accumulated during this period, a longer time period would provide a more comprehensive understanding on the treatment costs. Second, giving policy recommendations based on the results would be easier if the patient groups were comparable by factors such as disease stage. Third, the analysis is restricted to the costs incurred in the specialized medical care, ignoring, e.g., the economic impacts of sick leaves and the costs incurred in the primary health care. Finally, the relatively small sample size may bias the results as very high or low costs of individual patients get a relatively high weight in the data, as a result of which the median costs have been primarily emphasized when presenting the results.

## Conclusions

While the treatment of OPSCC can be based on either definitive (chemo)radiotherapy or primary surgical treatment, the prognosis of OPSCC is not significantly impacted by the chosen treatment modality. This enables to consider secondary factors, including treatment costs, when deciding on the treatment modality. This study shows that PST—with median total costs of 40 600€—is in Finland significantly more expensive than definitive RT and definitive CRT, the median total costs of which are 10 700€ and 13 300€, respectively. The present results differ from those previously reported in North American studies, which can be explained, e.g., by differences in the regulation of the health care systems as well as differences in the treatment practices.
